# Retraction: Application of LDA and word2vec to detect English off-topic composition

**DOI:** 10.1371/journal.pone.0283315

**Published:** 2023-03-15

**Authors:** The PLOS ONE Editors

Following the publication of this article [1], the corresponding author contacted the journal to request retraction.

Upon editorial follow up, similarities were noted between this article and a previous publication by another group [2]. The corresponding author has stated that the methods and part of the data in this article were provided by a third party and came from the previously published article [2].

In light of these issues, the *PLOS ONE* Editors retract this article [1].

All authors agreed with the retraction.

This article [1] reports modified material from [2], published 2018, Atlantis Press, which is offered under a CC-BY-NC license [3]. This retracted *PLOS ONE* article is therefore not offered under the Creative Commons Attribution License (CC-BY). At the time of retraction, the article [1] was republished to update its copyright statement. Readers should refer to the copyright notice in [2].

Update: Owing to an error, the Copyright statement on this article was not amended at the time of retraction. The retracted PLOS ONE article was removed from the PLOS ONE website on October 1, 2024 and the removed contents are no longer offered under the Creative Commons Attribution License. Readers should refer to [2] for the copyright notice.
